# 21-Deoxycortisone: A Novel Sensitive and Specific Newborn Screening Marker for Congenital Adrenal Hyperplasia

**DOI:** 10.3390/ijns12020027

**Published:** 2026-04-27

**Authors:** Mark de Hora, Natasha Heather, Dianne Webster, Benjamin B. Albert, Paul Hofman

**Affiliations:** 1 LabPlus, Auckland City Hospital, Auckland 1140, New Zealand; nheather@adhb.govt.nz (N.H.); diannew@adhb.govt.nz (D.W.); 2 Liggins’ Institute, University of Auckland, Auckland 1142, New Zealand; b.albert@auckland.ac.nz (B.B.A.); p.hofman@auckland.ac.nz (P.H.)

**Keywords:** congenital adrenal hyperplasia, newborn screening, bloodspot steroids, 21-deoxycortisol, 21-deoxycortisone

## Abstract

21-deoxycortisol is a sensitive and specific blood marker for congenital adrenal hyperplasia (CAH). We postulated that 21-deoxycortisone, the 11β-hydroxysteroid dehydrogenase metabolite of 21-deoxycortisol, may also be an accurate bloodspot marker of CAH. Measurement of 21-deoxycortisone was performed on 42 residual NBS specimens with a true positive result for CAH, 11 with a false negative result, and 439 specimens with a false positive result. For this study, the test was considered positive if 21-deoxycortisone was detected. The sensitivity and specificity of 21-deoxycortisone as a marker for classical CAH was calculated and compared to 21-deoxycortisol data from a previous New Zealand study. The method for 21-deoxycortisone measurement was linear to 1000 nmol/L and precision was 7.3–10.3%. The lower limit of quantification was 2 nmol/L, and recovery was 99%. 21-deoxycortisone was ≥2 nmol/L in all 42 true positive samples and in 10 false negative samples, and was not detected in the false positive group of specimens. The sensitivity of 21-deoxycortisone was 98.1%, and specificity was 100%. In a previous study, the sensitivity of 21-deoxycortisol was 88.7% and specificity was 99.8% in 1910 newborn screening tests carried out between 2018 and 2021. Incorporating 21-deoxycortisone into a second-tier test and adjustment of primary screening protocols could improve the accuracy of newborn screening for CAH. Longer term prospective studies on the performance of 21-deoxycortisone are warranted.

## 1. Introduction

Congenital adrenal hyperplasia is an autosomal recessive disorder characterised by a reduced synthesis of aldosterone and cortisol. Mutations in CYP21A2 result in reduced activity in 21-hydroxylase, an enzyme required for the synthesis of these essential steroid hormones. The lack of cortisol leads to a loss of negative feedback on the hypothalamus and pituitary, increasing adrenocorticotropin secretion, stimulating adrenal hyperplasia and steroidogenesis, which in turn is diverted by the metabolic block toward excessive androgen production [1]. Mineralocorticoid deficiency also occurs with more severe functional deficits in CYP21A2. Thus, there is a spectrum of disease depending on the severity of the mutation in CYP21A2. The severest form of CAH results from an almost complete absence in enzyme activity that can lead to life-threatening salt-wasting (SW-CAH) in the first weeks of life and progressive virilisation if not optimally managed over time. The simple virilising form (SV-CAH) has sufficient mineralocorticoid function to maintain electrolyte control under all but the most extreme conditions, but there is progressive virilisation during childhood [2]. The more common, non-classic form (NC-CAH) presents later in life with symptoms of androgen excess [3].

Newborn screening (NBS) for CAH has been available in New Zealand (NZ) since 1984 and prevents the life-threatening clinical complications that are associated with SW-CAH in the first few weeks after birth. Screening is performed by measurement of 17-hydroxyprogesterone (17OHP) on dried bloodspots collected by heel prick from babies 24–48 h after birth. Screening is sensitive for SW-CAH but is less sensitive for SV-CAH. Between 2005 and 2024, 1.2 million babies had NBS for CAH in NZ, and 49 cases of CAH were detected by screening. During that time, 11 babies with SV-CAH were not detected by screening and presented in early childhood with symptoms of androgen excess [4,5]. Children with classical CAH that were missed by screening may have benefitted from early detection and treatment.

Newborn screening has historically been challenging, with most programmes using 17OHP measured by immunoassay as the initial screening metabolite. Using 17OHP can result in many false positive tests, caused by elevated 17OHP measurements due to illness or prematurity [6]. Immunoassays are prone to antibody cross-reactivity, which causes falsely elevated measurements due to high peripheral concentrations of 17OHP precursors and associated sulphated conjugates that accumulate in premature babies [7]. More accurate screening is possible with the use of a second-tier test using liquid chromatography–tandem mass spectrometry (LC-MS/MS) that can measure multiple informative steroids simultaneously on the same bloodspot sample [8]. In NZ, the second-tier LC-MS/MS method incorporates 17OHP, androstenedione (A4), 21-deoxycortisol (21-DF) and cortisol (CORT). This approach has been successfully used in all newborn screening programmes in Australia and New Zealand, markedly improving the positive predictive value of CAH screening [9,10].

**Figure 1:**
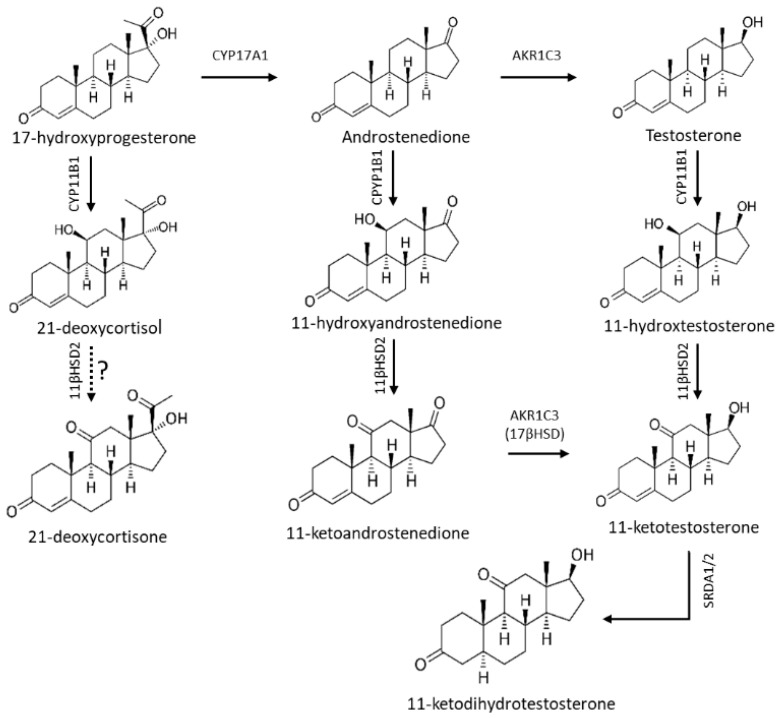
The 11-oxygenated steroid biogenesis pathway in congenital adrenal hyperplasia. Accumulating 17OHP overcomes the poor substrate affinity of 17-hydroxylase (CYP17A1) and is converted to androstenedione (A4), and then by aldo-keto reductase (AKR1C3) to testosterone (T). 17OHP, A4 and T are readily converted by adrenal specific 11β-hydroxylase (CYP11B1) to 21-deoxycortisol (21-DF), 11-hydroxyandrostenedione (11OHA4) and 11-hydroxytestosterone (11OHT). Further conversion by peripheral 11β-hydroxysteroid dehydrogenase type 2 occurs in the peripheral circulation to form 21-deoxycortisone (21-DE), 11-ketoandrostenedione (11KA4) and 11-ketotestosterone (11KT). 11KT is then converted at target tissues by 5α-reductase (SRDA1/2) to the potent androgen 11-ketodihydrotestosterone.

The use of LC-MS/MS in studies of adrenal steroidogenesis in health and disease has also identified novel blood markers of excessive adrenal androgen production in CAH. The 11-oxygenated androgens are the dominant 19 carbon steroids in adrenal hyperandrogenic conditions [11]. Accumulating A4 and testosterone are readily converted to 11-hydroxyandrostenedione and 11-hydroxytestosterone by adrenal specific 11-hydroxylase (CYP11B1), with further conversions to 11-ketoandrostenedione and 11-ketotestosterone by 11β-hydroxysteroid dehydrogenase type 2 (11βHSD2) in peripheral tissues [12]. Similarly, 21-DF is readily formed by CYP11B1 conversion of accumulating 17OHP in CAH, and is considered a sensitive and specific marker of 21-hydroxylase deficiency [13]. We postulated that 21-deoxycortisone (21-DE), the 11βHSD2 downstream metabolite of 21-DF, may also be a sensitive and specific blood marker for CAH (Figure 1).

In this study we compared the relative performance of 21-DF and 21-DE as newborn screening markers for CAH in New Zealand. The goal of the study was to determine whether the incorporation of 21-DE into a bloodspot LC-MS/MS second-tier method could inform improvements in the accuracy of NBS for CAH.

## 2. Materials and Methods

### 2.1. Newborn Screening Samples for 21-DE Measurement

For 21-DE measurement, 492 residual NBS specimens were included in the study. A false positive (FP) screening test for CAH was encountered in 439 specimens that were routinely collected by heel prick between 2015 and 2023. There were 53 residual NBS specimens from babies with CAH that were collected between 2005 and 2023, 42 of which had a true positive (TP) screen and 11 of which had a false negative (FN) screen. All FN cases presented in early childhood (3 months to 7 years) with symptoms of androgen excess. All specimens were collected between 1 and 49 days after birth. The birthweight (BW) recorded on specimens ranged between 464 g and 4250 g, with gestational ages between 23 and 41 weeks. Residual newborn screening samples are routinely stored, indefinitely, at a secure offsite facility at room temperature. Ethical approval for the study was granted by Auckland Health and Disability Ethics Committee (HDEC ethics reference 19/NTA/173).

Steroid-enriched whole bloodspots were used to calibrate and evaluate the performance of 21-DE measurement by LC-MS/MS. Details of bloodspot calibrator and control preparation have previously been described in detail [14]. There were six calibrators and 3 levels of control material used in the study.

### 2.2. Second-Tier Newborn Screening Data for 17OHP and 21-DF

Second-tier LC-MS/MS newborn screening data for 21-DF from 1910 NBS specimens, routinely collected from second-tier LC-MS/MS results, were included in the study. Samples had proceeded to second-tier testing when 17OHP by immunoassay was above the BW adjusted thresholds of ≥27 nmol/L in babies with a BW ≥ 1500 g and ≥37 nmol/L with BW < 1500 g. The second-tier test prior to December 2017 was a repeat 17OHP immunoassay after solvent extraction to remove interfering polar steroid conjugates. The second-tier immunoassay was replaced by a LC-MS/MS method to measure 17OHP, A4, 21DF and CORT. For both the immunoassay and LC-MS/MS second-tier tests, a test was considered positive if 17OHP ≥ 23 nmol/L (≥34 nmol/L if BW < 1500 g). A group of screening studies were then used to develop a new laboratory protocol in 2022 that incorporated new thresholds for 17OHP and the introduction of a (17OHP + A4)/CORT ratio and 21-DF as additional second-tier markers [5,10]. Since 2022, regardless of the second-tier LC-MS/MS 17OHP and (17OHP + A4)/cortisol result, a 21-DF > 2 nmol/L is considered a positive test.

To improve the power of the study, 17OHP and 21-DF data from 37 retrospective NBS specimens from babies with CAH collected between 2005 and 2023 were included. The ratio (17OHP + A4)/cortisol was not available in the retrospective NBS specimens as CORT is not stable in dried bloodspots that are stored for long periods at room temperature [15]. Data for 17OHP and 21-DF collected from 36 retrospective samples had already been reported in a previous NZ study [10]. Measurement of 21-DE was carried out in all 36 retrospective samples.

### 2.3. LC-MS/MS Analysis

The bloodspot sample preparation and LC-MS/MS method for expanded steroid profiling that included 21-DE has previously been described in detail [14]. Briefly, two 3 mm bloodspots were punched into a deep well microtiter plate. Then, 20 µL of methanolic internal standard containing deuterated 21-deoxycortisol (21-DF-d8) and 200 µL of extraction solution (80% acetonitrile) was added to each well. The plate was mixed for 30 min and 200 µL of the extraction solution was dried in a 1.5 mL high recovery glass vial and reconstituted with 80 µL of 40% methanol for LC-MS/MS analysis. The multiple reaction monitoring signals used for quantitation were *m*/*z* 345.2 > 163.2 for 21-DE, and *m*/*z* 355.2 > 319.1 for 21-DF-d8. Calibration and quantitation were performed by plotting calibrator values against the peak area ratio 21-DE/21-DF-d8.

### 2.4. LC-MS/MS Performance and Data Analysis

Statistical analysis was performed using Graphpad PRISM (San Diego, CA, USA) version 7.02 statistical software and Microsoft Excel. LC-MS/MS method linearity was assessed with serial dilutions of a steroid enrichment solution, containing 21-DE, that was used to enrich bloodspots for method evaluation. Precision was assessed with control material (2 levels), either within a single calibration (*n* = 10) or between calibrations (*n* = 10). Evaluation samples enriched with known quantities of steroid material was used to assess recovery. The lower limit of quantification (LLOQ) was defined as the lowest observed concentration with an inter-batch precision of <30% (*n* = 10) in the evaluation samples. The criterion for LLOQ was chosen as a previous study found that the concentrations of some analytes measured in a peripheral 3 mm punch blood disc can be between 25 and 30% higher than a 3 mm punch obtained from a central position [16].

Receiver operating characteristic plots (ROC) were constructed for 21-DE and 21-DF data and compared. For the ROC plots, data for TP and FN were combined and plotted against the FP group. The area under the curve (AUC) of the ROC plot represents the probability that a randomly chosen sample will correctly rank the respective steroid measurement of a TP/FN specimen higher than a chosen FP sample. The sensitivity and specificity of 21-DF and 21-DE measurements were calculated and compared.

## 3. Results

The method for 21-DE measurement was linear beyond 1000 nmol/L (R^2^ > 0.995). Within-batch co-efficient of variation (CV) was 13.8% at a mean concentration of 4 nmol/L and 9.9% at a mean concentration of 31 nmol/L. The between-batch CV was 10.3% at 4 nmol/L and 7.3% at 31 nmol/L. Accuracy was assessed by calculating the recovery of known enrichment values for bloodspots, and 21-DE recovery was 99%. The LLOQ was 2 nmol/L with a within-batch variation of 19%. There was full chromatographic resolution between 21-DF and 21-DE.

In the cases included in the study, all TP specimens proceeded to second-tier testing while 4 of 11 cases missed by screening underwent second-tier immunoassay analysis when screening was performed.

In residual NBS specimens, retrospective measurement of 21-DE by LC-MS/MS was ≥2 nmol/L in all 42 TP specimens (median 130 nmol/L, range 20–602, Appendix A) and in 10 of 11 FN samples (median 20 nmol/L, range 0–54, Table 1). The sensitivity of 21-DE was 98·1%. 21-DE was not detected in any of the FP samples when it was measured retrospectively in 439 FP specimens (specificity = 100%).

As a comparator, 21-DF data from a previously published NZ screening study [5] revealed that 21-DF was ≥2 nmol/L in 34 of 36 TP samples (median 22 nmol/L, range 0–209) and ≥2 nmol/L in 5 of 10 FN (2006–2015) specimens (median 3 nmol/L, range 0–13). The sensitivity of 21-DF to detect CAH in the specimen group was 84.8%, while 21-DF was above the LLOQ in 4 out of 1909 s-tier specimens (3–5 nmol/L) with a specificity of 99.8% (Table 2).

**Figure 2:**
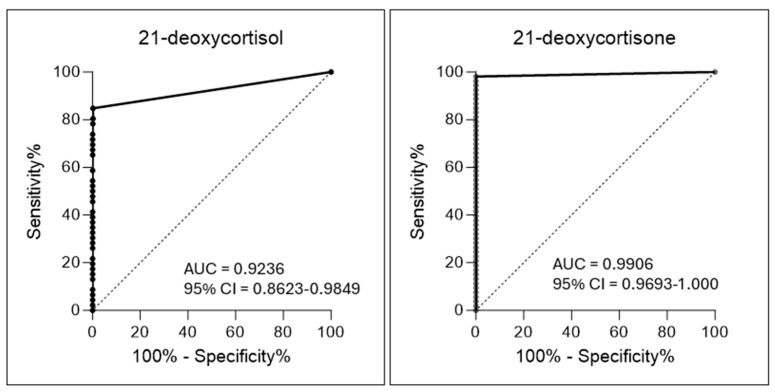
Receiver Operating Characteristic Curves for 21-DE and 21-DF for samples included in the Study. AUC: Area under the curve. The dashed line indicates the line of no discrimination between CAH and non-CAH specimens.

For 21-DE, the AUC of the ROC plot was 0.9906 compared to an AUC for 21-DF of 0.9236 for the specimens included in the study (Figure 2). The data from this study would indicate that 21-DE is a more accurate NBS marker for CAH in the newborn period.

## 4. Discussion

We have further developed a second-tier test for newborn screening for CAH that facilitates the incorporation of 21-deoxycortisone as an additional sensitive and specific marker. The method is sufficiently precise, and recovery experiments indicate that the method is sufficiently accurate for use, although no external quality assurance or bloodspot reference material was available for use in method evaluation. Our study found that the sensitivity and specificity of 21-DE was higher than 21-DF when used as a bloodspot marker for CAH and therefore may be a useful single or complimentary screening marker.

The limitations of bloodspot 17OHP immunoassays in NBS for CAH is well documented. Immunoassay interferences caused by high concentrations of 17OHP precursors can lead to poor screening specificity in premature babies [17,18]. In addition, we have previously shown that 17OHP measurements by LC-MS/MS have a negative correlation with BW and GA and there is an overlap in 17OHP between TP and FP measurements [19]. As 21-DF, the CYP11B1 metabolite of 17OHP, is rarely detectable by LC-MSMS, it is possible that there is an extra adrenal source of 17OHP in premature and sick neonates. Newborns, particularly premature neonates have elevated concentrations of 17-hydroxypregnenolone that may then be converted to 17OHP by 3β-hydroxysteroid dehydrogenase activity in the liver [20] that can lead to false positive screening tests. Adrenal specific markers such as 21-DF and 21-DE for NBS for CAH have advantages over 17OHP as accurate single markers.

21-DF is a highly sensitive and specific newborn screening marker for CAH. The results of a 2-year evaluation of newborn screening in the Netherlands revealed elevated 21-DF levels in 15 classical TP CAH cases and in 5 specimens from babies with NC-CAH that were considered for FP screening tests, as well as a further specimen with elevated 21-DF from a premature baby that did not have any CAH mutations [21]. The study highlighted the high sensitivity of 21-DF, and consistent with studies from New Zealand, demonstrated that babies who do not have CAH can also have mildly elevated 21-DF during the newborn period. In contrast, a previous New Zealand NBS study did not detect 21-DF in all TP specimens and identified elevated 21-DF in several FP samples [5], which indicates that an additional complimentary marker may improve screening accuracy.

Based on our data, the introduction of LC-MSMS will improve screening sensitivity as 4 of the 10 FN samples that had proceeded to a second-tier immunoassay would have been detected using a second-tier LC-MS/MS approach. In this study, 21-DE was detected in all TP specimens and in all but one FN samples, and was not detected in any FP samples. This suggests that 21-DE is a more sensitive and specific marker than 21DF and could replace 21-DF or be added as a complimentary marker for accurate screening. The risk, however, with evidence from the Netherlands experience [21], is that cases of NC-CAH, which is not a screening target in either New Zealand or the Netherlands, may also be detected using these sensitive markers, and patients with NC-CAH are unlikely to benefit from early treatment. Without CYP21A2 mutation analysis to specifically diagnose which positive cases have NC-CAH mutations, there is a risk that unnecessary or excessive glucocorticoid treatment will be given to children with NC-CAH and lead to worse outcomes than if they were not identified in the newborn period.

In New Zealand, two birthweight-adjusted thresholds are applied for the primary newborn screening 17OHP immunoassay. We have previously shown, along with other studies, that there is a negative correlation between BW and GA with 17OHP immunoassay and LC-MS/MS measurements [19]. Our data suggested that multiple BW or GA thresholds for 17OHP immunoassays may be appropriate to improve screening sensitivity for classical CAH and would reduce the burden of second-tier testing on the screening laboratories. We had previously shown that lowering 17OHP immunoassay thresholds in babies born at term to 22 nmol/L (97.5th centile in NZ) in babies ≥ 2500 g would have resulted in additional FN specimens proceeding to second-tier testing that may result in improved screening sensitivity [19], and this is borne out by the LC-MS/MS results in the FN cases that were included in this study. A combination of 21-DF and 21-DE would then distinguish between CAH and non-CAH specimens with a high level of accuracy.

A limitation in our study is the retrospective nature of 21-DE measurement in samples stored over long periods. Ten of the FN specimens were stored for 10–20 years at room temperature under controlled conditions. A previous study demonstrated that CORT and 11-deoxycortisol, both 11-hydroxylated steroids, were not stable when stored for long periods, although 21-DF, 17OHP and A4 were stable for at least 1 year [16]. While longer term studies are not available, this suggests that some steroids degrade on room temperature storage. While our 21-DE measurements were sufficient for this study, we did not detect 21-DE in a FN sample that was in storage for almost 20 years. Short-term stability studies with manufactured bloodspots enriched with 21-DE are warranted, and long-term studies with data collected prospectively in screening samples would be beneficial, but may require international collaboration.

Despite the limitations of 17OHP, all international NBS programmes use 17OHP as a primary screening test. Worldwide, significant cost reduction and efficiencies could be achieved if a primary screening test measuring 21-DF or 21-DE was available. Several studies have reported on the performance of 21-DF immunoassays in plasma that can distinguish between classical CAH, non-classical CAH, and controls, and that 21-DF measurement by immunoassay is a sensitive and reliable diagnostic test [22]. There has already been a call to replace 17OHP as a primary screening marker [23]. Developing and implementing a sufficiently accurate immunoassay in dried bloodspots for 21-DF or 21-DE would offer significant improvements to screening programmes, including limiting the need for the expensive LC-MS/MS equipment that is currently required for accurate screening.

## Figures and Tables

**Table 1:** Bloodspot 17OHP and 2nd-tier immunoassay and retrospective LCMSMS results on classical CAH cases reported to the NZ NBS programme that were missed (false negative) by screening, 2006–2023.

Year	1st Tier 17OHP nmol/L	2nd Tier 17OHP nmol/L	Retrospective 21-DF nmol/L	Retrospective 21-DE nmol/L
2023	24	-	3	21
2015	5	-	<2	7
2015	26	-	10	54
2014	30	17	5	29
2012	13	-	<2	7
2010	72	19	13	34
2010	34	17	7	32
2010	6	-	<2	4
2009	30	21	11	31
2008	19	-	<2	5
2006	11	-	<2	<2

**Table 2:** Sensitivity and specificity of bloodspot 21-DF and 21-DF to distinguish between babies with classical CAH and babies that did not have CAH.

	21-Deoxycortisol	21-Deoxycortisone
Sensitivity	94.7%	98.1%
Specificity	99.8%	100%

## Data Availability

The data presented in this study is available on request from the corresponding author, through application to the Prevention Directorate of the New Zealand Ministry of Health.

## Abbreviations

The following abbreviations are used in this manuscript:

CAH	Congenital Adrenal Hyperplasia
21-DF	21-deoxycortisol
21-DE	21-deoxycortisone
NBS	Newborn Screening
TP	True Positive
TN	True Negative
FN	False Negative
FP	False Positive
SW-CAH	Salt wasting CAH
SV-CAH	Simple Virilising CAH
NC-CAH	Non-Classical CAH
17OHP	17-hydroxyprogesterone
LC-MS/MS	Liquid Chromatography Tandem Mass Spectrometry
A4	Androstenedione
T	Testosterone
CYP11B1	11-hydroxylase
NZ	New Zealand
CORT	Cortisol
11βHSD2	11β-hydroxysteroid dehydrogenase type 2
CYP21A2	21-hydroxylase
BW	Birthweight
LLOQ	Lower limit of quantification
ROC	Receiver operating curve
AUC	Area under the curve

## Appendix A

**Table A1:** Newborn Screening Data for True Positive CAH cases in NZ 2005–2023.

No	Year	Sex	Age Dats	BW Grams	1st Tier 17OHP nmol/L	2nd Tier17OHP nmol/L	21-DF nmol/L	21-DE nmol/L
1	2023	M	2	3780	391	229	113	601
2	2022	F	5	3300	440	232	64	256
3	2022	M	2	2820	534	443	17	105
4	2022	M	20	3280	412	335	53	228
5	2022	F	1	4190	314	110	20	87
6	2022	F	3	3750	168	154	12	59
7	2021	F	3	2450	599	301	29	262
8	2021	M	2	3655	715	462	32	140
9	2020	M	1	4060	667	1422	88	228
10	2020	F	2	2960	627	551	<2 ^a^	225
11	2020	F	2	2560	368	128	64	457
12	2019	M	2	2902	250	112	22	158
13	2019	M	2	3810	613	383	39	134
14	2019	F	2	3890	686	833	73	382
15	2018	M	2	3670	670	761	64	290
16	2018	M	2	3975	434	138	25	139
17	2017	M	2	3010	232	139	8	228
18	2016	M	30	3700	458	505	385	602
19	2016	F	2	3560	416	420	18	167
20	2014	F	3	3750	319	93	9	20
21	2014	M	3	4330	401	326	12	115
22	2013	F	1	4200	242	211	188	284
23	2013	F	3	3610	209	179	12	60
24	2012	F	1	4100	108	56	13	58
25	2012	M	4	3165	69	53	9	30
26	2012	F	2	3650	211	160	31	86
27	2011	M	3	2970	207	142	14	126
28	2010	F	- ^b^	- ^b^	144	111	27	77
29	2010	F	1	2410	295	239	35	114
30	2010	F	1	3710	305	131	49	114
31	2009	F	5	3200	301	201	69	159
32	2009	M	2	1810	354	305	112	297
33	2008	M	2	3350	255	186	12	78
34	2008	M	3	4340	282	63	28	63
35	2008	F	4	3750	135	66	3	30
36	2008	M	5	4108	419	201	33	87
37	2008	F	2	3605	326	267	88	170
38	2007	M	2	4330	415	185	28	98
39	2006	M	3	3620	404	575	21	89
40	2005	F	4	3760	583	569	153	270
41	2005	F	1	3150	127	67	26	50
42	2005	F	2	3340	444	285	26	119

^a^ Case number 10 was considered a TP result due to the elevated (17OHP + A4)/cortisol that is incorporated in laboratory protocol for screening. ^b^ data was not provided on the newborn screening specimen.
